# ACVR1 mutation and Fibrodysplasia Ossificans Progressiva in Chinese children

**DOI:** 10.1186/1546-0096-9-S1-P30

**Published:** 2011-09-14

**Authors:** Assunta CH Ho, Joannie Hui, KH Chan, KL Liu, Bobby KW Ng, Ivan FM Lo, STS Lam

**Affiliations:** 1 Department of Pediatrics, Prince of Wales Hospital, Chinese University of Hong Kong, Hong Kong SAR, Hong Kong; 2 Clinical Genetic Service, Department of Health, Hong Kong SAR, Hong Kong; 3 Department of Orthopedics and Traumatology, Prince of Wales Hospital, Chinese University of Hong Kong, Hong Kong SAR, Hong Kong

## Introduction

Fibrodysplasia Ossificans Progressiva (FOP) is a rare autosomal dominant connective tissue disorder characterized by congenital great toes malformation and progressive heterotopic osteogenesis leading to progressive debilitating ankylosis of the body. A recurrent missense mutation in the activin receptor 1A/activin-like kinase 2 (ACVR1/ALK2), a bone morphogenetic protein (BMP) receptor, was identified in almost all FOP patients^1^. Here we report two unrelated Chinese FOP adolescents harbouring this mutation. Both were referred to the rheumatological service with joint stiffness and abnormal calcification on imaging.

## Case 1

CSNM developed progressive jaw ankylosis since twelve years of age. She started to have recurrent soft tissue swellings involving neck, shoulders and back at sixteen. At that time she was suffering from pulmonary tuberculosis. The swellings subsided spontaneously, leaving bony hard masses.

## Case 2

WCY was born with multiple congenital abnormalities including hypospadias, atrial septal defect (ASD) and multiple exostoses. Starting from twelve year of age, he developed ankylosis of the left temporomandibular joint. The first FOP flare started with a painful swelling over his back and neck. This was followed by stiffening of the muscles.

Both patients had a heterozygous c.617G >A mutation detected in the exon 7 of the ACVR1 gene, resulting in an Arg206His substitution.

## Conclusion

Confirmation of the diagnosis of FOP is relatively more straightforward now with the availability of molecular genetic testing. A high index of suspicion is the key to diagnose these patients early with unexplained recurrent ossified swellings and congenital feet abnormalities. Unnecessary and often harmful biopsy procedures could be avoided during the diagnostic workup of these patients.

**Fig 1:**
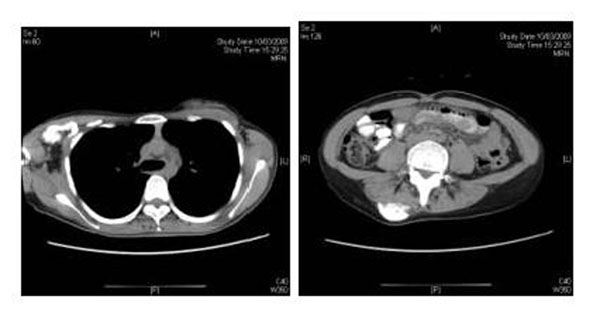
CT images of case 1- extraosseous ossification

**Fig 2:**
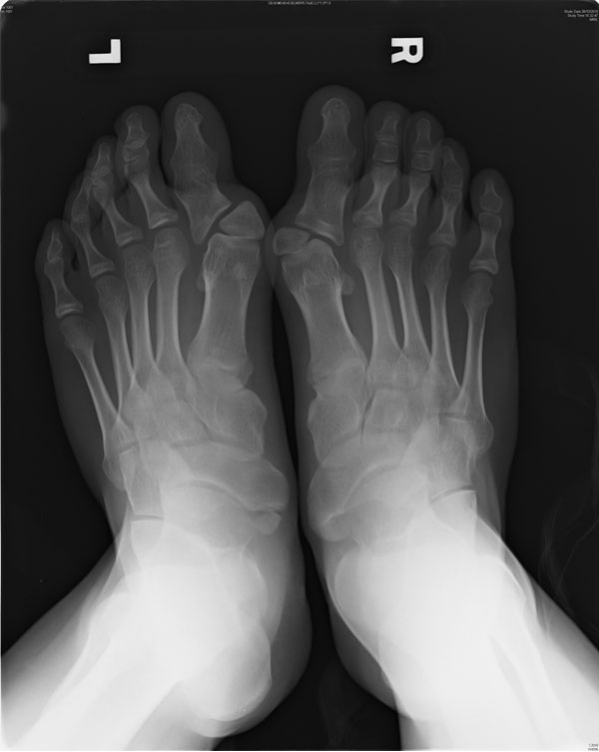
great toe abnormality of case 2

## References

[B1] Shore EM Xu M Feldman GJ A recurrent mutation in the BMP type I receptor ACVR1 causes inherited and sporadic fibrodysplasia ossificans progressive Nat Genet 2006 28 525 527 10.1038/ng1783 16642017

